# Angioma serpiginosum in zosteriform distribution on abdomen: A rare presentation

**DOI:** 10.1002/ccr3.3995

**Published:** 2021-03-02

**Authors:** Kamal P. Acharya, Prajwal Pandey, Rajan Shah, Muna Bista, Samir Shrestha

**Affiliations:** ^1^ Department of Dermatology and Venereology B.P. Koirala Institute of Health Sciences Dharan Nepal; ^2^ Department of Pathology B.P. Koirala Institute of Health Sciences Dharan Nepal; ^3^ Sindhuli District Hospital Dharan Nepal

**Keywords:** angioma serpiginosum, nevoid disorder, serpiginous, zosteriform

## Abstract

Angioma serpiginosum is a rare benign nevoid disorder affecting the small vessels of the upper dermis. We are presenting a case of this rare disease in an 11‐year‐old girl who presented with this condition on abdomen in zosteriform pattern which is rare presentation and is the first of its type.

## Graphical abstract

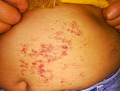

## 1 INTRODUCTION

Angioma serpiginosum is a rare benign nevoid disorder affecting the small vessels of the upper dermis. We are presenting a case of this rare disease in an 11‐year‐old girl with multiple progressive asymptomatic scattered and confluent punctate erythematous to violaceous macules over the left side of the abdomen in T8‐T10 dermatomal distribution with normal surrounding and intervening skin since birth. Histopathological examination (HPE) revealed few ectatic congested thin‐walled blood vessels in papillary dermis with few areas of downward growth of the rete ridges between these blood vessels. Based on the clinical and the HPE, a diagnosis of angioma serpiginosum was made. This rare vascular anomaly and abdomen involvement with zosteriform distribution are rarely reported in the literature.

Angioma serpiginosum is a rare benign primary telangiectatic disorder affecting the capillaries of the upper dermis characterized by asymptomatic pinpoint macules that group together in linear, serpiginous, or gyrate patterns.^1^, ^2^ It usually starts in childhood or early adolescence and has female predominance. Most cases are sporadic; however, few cases may be familial. The exact pathophysiology of this condition is unknown. It is more common in the extremities and gluteal region. There are few case reports of the condition involving palms, soles, mucosa, and chest. However, this is the first case report of the condition involving the abdomen.

## 2 CASE PRESENTATION

An 11‐year‐old girl presented with multiple asymptomatic erythematous lesions on the left abdomen which was first noticed by her mother as multiple red pinhead‐sized lesions during birth. The lesions progressively extended with new satellite lesions appearing around the previous ones with the increasing age. There was no family history of similar skin lesions. There were no visual or neurological symptoms.

**FIGURE 1:**
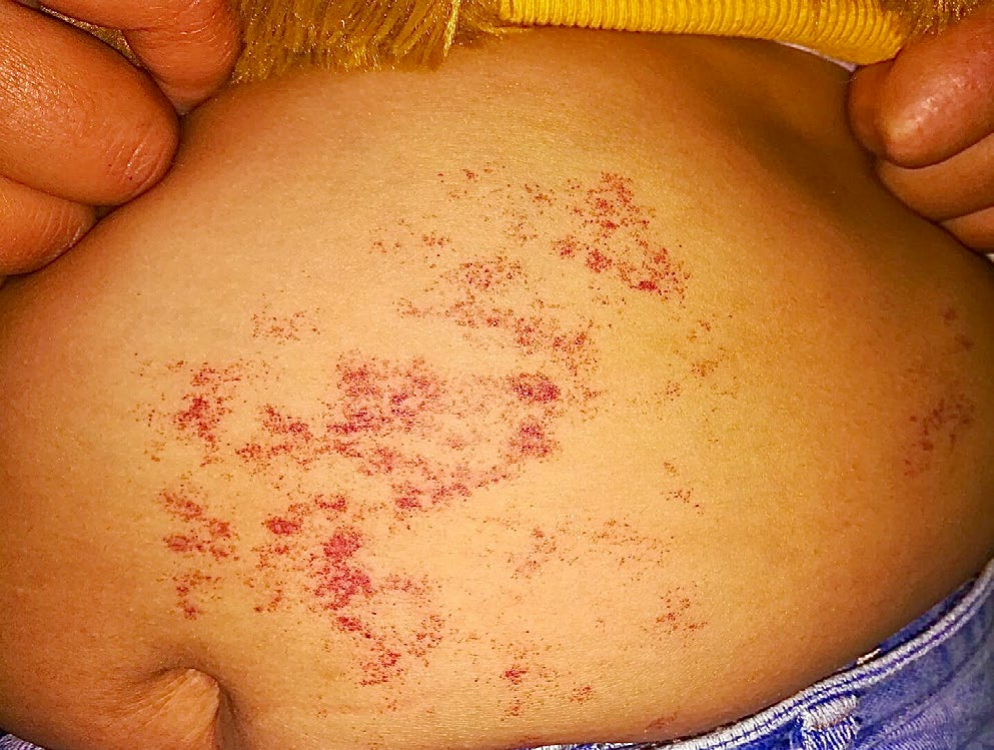
Multiple scattered and confluent punctate erythematous to violaceous macules over the left side of the abdomen in T8‐T10 dermatomal distribution

Cutaneous examination revealed multiple discrete and confluent punctate erythematous to violaceous macules on the left abdomen in T8 to T10 dermatomal distribution with normal surrounding and intervening skin (Figure 1). There was no similar lesion in other parts of the body. Her general physical and systemic examination was unremarkable.

**FIGURE 2:**
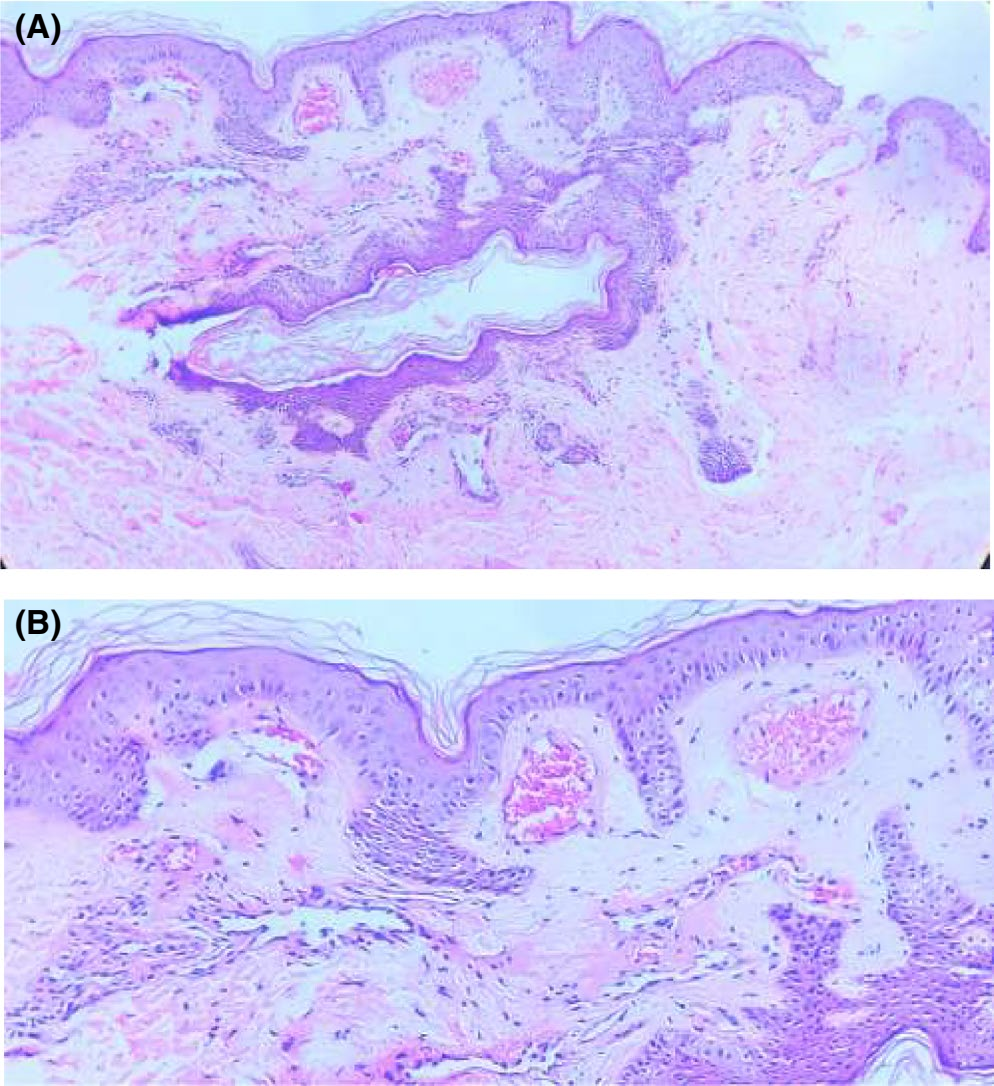
A, Section shows epidermis and dermis with papillomatosis and focal elongation of rete ridges. Hematoxylin and eosin stain (original magnification 10X). B, Basket weaves hyperkeratosis, papillomatosis, hypogranulosis and pigmented basal layer. Papillary dermis showed few ectatic congested thin walled blood vessels. Few areas showed downward growth of the rete ridges between these blood vessels. Hematoxylin and eosin stain (original magnification 40X

Skin biopsy taken from the lesion showed epidermis with basket weave hyperkeratosis, papillomatosis, and hypogranulosis. Papillary dermis showed few ectatic congested thin‐walled blood vessels. A few areas showed downward growth of the rete ridges between these blood vessels. There was no epidermal acanthosis and extravasation of RBCs, inflammatory cell infiltration, and hemosiderin deposition were not seen in the dermis (Figure 2A,B). Based on the clinical and histopathological findings (HPE), a diagnosis of angioma serpiginosum was made.

The patient and her parents were counseled about the benign nature of the disease and planned for laser therapy if any cosmetic concerns in the future.

## 3 DISCUSSION

Angioma serpiginosum (AS) is a rare vascular disorder described first by Hutchinson in 1889 as a “serpiginous or infective nevus” and then named by Crocker in 1894.^2^, ^3^ Cases are usually sporadic; however, familial cases with an autosomal‐dominant or X‐linked‐dominant inheritance and few cases with PORCN gene mutation or deletion have been reported.^4^ Angioma serpiginosum results from the proliferation of endothelial cells resulting in dilated capillaries. There are various theories regarding its pathogenesis. One of the theories suggest the role of estrogen while another theory calling for the role of cold temperature in its pathogenesis. However, both of the theories are not accepted universally.^7^, ^8^

The condition usually starts in childhood or birth with a female predominance.^5^, ^6^ This condition presents as asymptomatic multiple, pinpoint violaceous or erythematous macules, nonblanching and clustered in an area or form large sheets distributed in serpiginous, and linear (Blaschkoid distribution) or annular pattern.^1^ Lesions are typically unilateral and located predominantly on the lower limbs and extremities but can be extensive.^5^, ^9^ However, palms, soles, mucosal, and truncal involvement is rare. A few cases with segmental truncal and mono‐lateral plantar area involvement have been reported.^6^, ^8^, ^10^, ^11^, ^12^, ^13^ This condition progresses slowly and usually attains stability at puberty with some partial spontaneous resolution in late adulthood. The diagnosis is usually made clinically and is confirmed by histological findings of distended ectatic capillaries lined by flattened endothelium cells of normal appearance and the absence of inflammation, erythrocyte extravasations, and hemosiderin deposition.^14^ Treatment is only indicated for cosmetic reasons for which long‐pulsed 1064 nm Nd:YAG vascular laser is the best option.^15^

In our case, the patient presented with localized telangiectasia without skin atrophy. The differentials for the condition include Angioma serpiginosum, capillary malformation, unilateral nevoid telangiectasia, and purpuric conditions.^16^ Presence of the lesions since birth as unilateral, asymptomatic erythematous, pinpoint to pinhead‐sized nonblanchable macules and papules in the zosteriform pattern (T8‐T10) without atrophy and perilesional halo supports the diagnosis of angioma serpiginosum. The histological findings confirmed the diagnosis. The peculiarity about our case is the site of lesion. This is the first case as per our knowledge with presentation of the lesion solely on abdomen. In our case, the parents were not concerned about the lesions, so no treatment was given and asked for follow‐up later in case of cosmetic concerns.

## CONFLICT OF INTEREST

None.

## AUTHOR CONTRIBUTIONS

KA: concept, manuscript preparation, and literature search. PP: manuscript editing, guidance, and final approval. RS: histopathological reporting. MB: manuscript preparation and literature search. SS: manuscript editing and correspondence Author.

## ETHICAL APPROVAL

Consent obtained from the parents of the patient for publication and use of the images.

## Data Availability

We agree to make the manuscript available to general people and are also ready to provide other necessary data regarding the manuscript in case required.
